# Impact of combined versus single-mode training programs based on drop jump and specific multidirectional repeated sprint on bio-motor ability adaptations: a parallel study design in professional basketball players

**DOI:** 10.1186/s13102-022-00551-w

**Published:** 2022-08-24

**Authors:** Seifeddine Brini, Daniel Boullosa, Julio Calleja-González, Daniel J. van den Hoek, Hadi Nobari, Filipe Manuel Clemente

**Affiliations:** 1 Research Unit, Sportive Performance and Physical Rehabilitation, High Institute of Sports and Physical Education of Kef, University of Jendouba, Kef, Tunisia; 2 Integrated Institute of Health, Federal University of Mato Grosso Do Sul, Campo Grande, Brazil; 3 College of Healthcare Sciences, James Cook University, Townsville, Australia; 4 Physical Education and Sport Department, Faculty of Education and Sport, University of the Basque Country (UPV/EHU), 01007 Vitoria-Gasteiz, Spain; 5 Faculty of Kinesiology, University of Zagreb, 10000 Zagreb, Croatia; 6 School of Behavioral and Health Sciences, Faculty of Health Science, Australian Catholic University, Brisbane, Australia; 7 Department of Exercise Physiology, Faculty of Educational Sciences and Psychology, University of Mohaghegh Ardabili, Ardabil, 56199-11367 Iran; 8 Department of Motor Performance, Faculty of Physical Education and Mountain Sports, Transilvania University of Braşov, 500068 Braşov, Romania; 9 Faculty of Sport Sciences, University of Extremadura, 10003 Cáceres, Spain; 10 Escola Superior Desporto E Lazer, Instituto Politécnico de Viana Do Castelo, Rua Escola Industrial eComercial de Nun’Álvares, 4900-347 Viana do Castelo, Portugal; 11 Research Center in Sports Performance, Recreation, Innovation and Technology (SPRINT), 4960-320 Melgaço, Portugal; 12 Instituto de Telecomunicações, Delegação da Covilhã, 1049-001 Lisbon, Portugal

**Keywords:** Athletic performance, Physical fitness, Team sports, Agility, Fatigue, Muscle strength, Stability

## Abstract

**Background:**

Jumping and specific multidirectional repeated sprint ability are important in basketball. The objective of this study was to assess the contributions of 8-week combined versus single-mode training programs based on drop jump (DJ) and specific multidirectional repeated sprint (MRSA) on repeated sprint ability performances, body balance and lower limbs power in male professional basketball players.

**Methods:**

This study followed a randomized parallel study design. Fifty-two professional male basketball players from the Tunisian first division participated in this study. The players were randomly assigned to 4 groups: DJ group (JG; n = 13), MRSA group (RSG; n = 13), combined group (COMB; n = 13) and an active control group (CON; n = 13). The JG, RSG and COMB groups completed the 8-week training programs with 2 sessions per week while the CON continues their regular basketball training. Training volume was similar between groups all over the experimental period. Before and after the intervention, the four groups were evaluated for the stork test, Y-balance test, the repeated sprint ability test (IRSA_5COD_), the squat jump (SJ) and countermovement jump (CMJ) tests, the single leg drop jump test, the five time-jump test and T—change of direction (CoD) test.

**Results:**

All measures displayed significant main effect, (medium/small) magnitude (effect size) improvements for time (post-test > pre-test) except the physiological parameters for IRSA_5COD_. Significant time × group interactions were revealed for body balance, *T* test, IRSA_5COD_ (total time and best time) and jump tests (vertical/horizontal). Bonferroni corrected post-hoc tests revealed significant greater improvement in favor of RSG and COMB compared to JG for body balance, CoD and IRSA_5COD_. Moreover, greater improvement in CMJ, SJ and single leg DJ in favor of JG compared to the RSG. In addition, a greater CoD improvement was observed in favor of COMB when compared to the RSG.

**Conclusion:**

Combined and single-mode training programs based on DJ and MRSA contributed to a significantly better performance in specific basketball physical fitness parameters with results favoring combined interventions.

**Supplementary Information:**

The online version contains supplementary material available at 10.1186/s13102-022-00551-w.

## Background

Basketball is a court-based team-sport that requires high-intensity intermittent forwards, backward, and lateral high-speed movements during games [i.e., sprints with change of direction (CoD)] [1, 2]. Additionally, time-motion analyses have shown that basketball players change movement types every 1–3 s during a game [3]. In the same context, Caprino et al. [4] reported that repeated sprint sequences with CoD are frequently followed by other actions such as vertical jumps (jump shots, lay-ups, blocks) that are integral in match outcomes. Thus, high CoD and jump performances are considered a particularly critical physical demand in basketball players [5, 6].

Considering jumping and specific multidirectional repeated sprint ability (MRSA) are important in basketball, it has been recommended to use both plyometric jump training and MRSA to improve muscular strength and power, to decrease the incidence and severity of sport-related injuries and to optimize basketball specific performances [7, 8]. Concerning MRSA training, previous studies reported that this kind of training regimen based on CoD repeated sprint may improve some specific aspects of basketball-related physical fitness including vertical jump and CoD performances [7, 9, 10]. Moreover, Kibele et al. [11] reported that rapid CoD challenges the ability to either maintain or return the center of gravity over the base of support (metastability) and thus provides a stress to dynamic balance. Additionally, rapid CoD repeatedly shifts the center of gravity outside the base of support and challenges the equilibrium or metastability [11]. Thus, MRSA training may be a useful training strategy to improve body balance in basketball players especially that balance leads basketball player to a better body control.

On the other hand, several studies showed the positive effect of plyometric jump training for improving some specific physical fitness performances (i.e., jumping, sprinting, repeated sprint ability (RSA) and CoD) in team sports generally and basketball more specifically [8, 12–14]. In the same context, Matavulj et al. [12] reported that a training program including a drop jump (DJ) protocol led to a better vertical jump and maximal voluntary force production in elite basketball players. Recently, Zagatto et al. [15] suggested that a DJ protocol may significantly improve RSA in basketball players. Additionally, several studies showed that plyometric training as a dynamic form of resistance training with a rapid stretch shortening cycle (SSC), involving both vertical and horizontal displacements of the individual’s center of gravity may stimulate body balance and help players to control their body position [13, 14, 16]. As such, plyometric training based on DJ exercises may be a useful training protocol to improve body balance in basketball players. Nonetheless, due to the rules, limited playing space, and the demands of the game, players need a combination of strength, power and CoD ability to win a running or jumping duel, and to grasp the ball before an opponent. Thus, it will be important to evaluate new training methods replicating the real game demands.

Otherwise, a few recent investigations in soccer and handball have examined the impact of combined plyometric and change of direction training programs and have reported a significant improvement in body balance, CoD, jump and RSA performances [17–19]. However, to the best of our knowledge, this hypothesis has not been tested yet in basketball.

Therefore, the aims of this study were to examine the contributions of 8-week combined versus single-mode training programs based on DJ and MRSA on repeated sprint performances, body balance and lower limbs power in professional basketball male players. Considering previous literature [7, 8, 15, 18], we hypothesized that the combined and the single mode training program based on DJ and MRSA would significantly affect body balance, jump, CoD and RSA performance with a synergistic effect following combined DJ and MRSA training.

## Methods

### Study design and setting

During the present study, adaptations following combined versus single-mode training programs based on DJ and MRSA were assessed using a parallel group randomized study design that included pre- and post-testing and three training interventions in between. Participants were randomly assigned either to an active control group (CON) or to one of three experimental groups: DJ training group (JG), MRSA training group (RSG), and combined training group (COMB). The randomization process was conducted using randomly permuted blocks using Research Randomizer [20, 21], a program published on a publicly accessible website (http://www.randomizer.org). Two independent researchers generated the random allocation sequence, enrolled participants, and assigned participants to the intervention groups [21].

The current study was conducted at the beginning of the competitive season between October and December of 2021. Overall, the study lasted 11 weeks. During the experimental period, participants trained five times per week and completed one game per week during the weekend. JG, RSG and COMB groups completed 8 weeks DJ, MRSA and combined training respectively (see details below) with a frequency of two sessions per week (Tuesday and Thursday) while the CON continued their regular training program. Training volume was similar between all groups over the experimental period. No additional exercises of strength were conducted by any of the experimental groups [7]. Before and after the 8-week training intervention the four groups were tested to detect adaptations on body balance, repeated sprint ability performances and lower limbs power.

### Participants

**Fig. 1:**
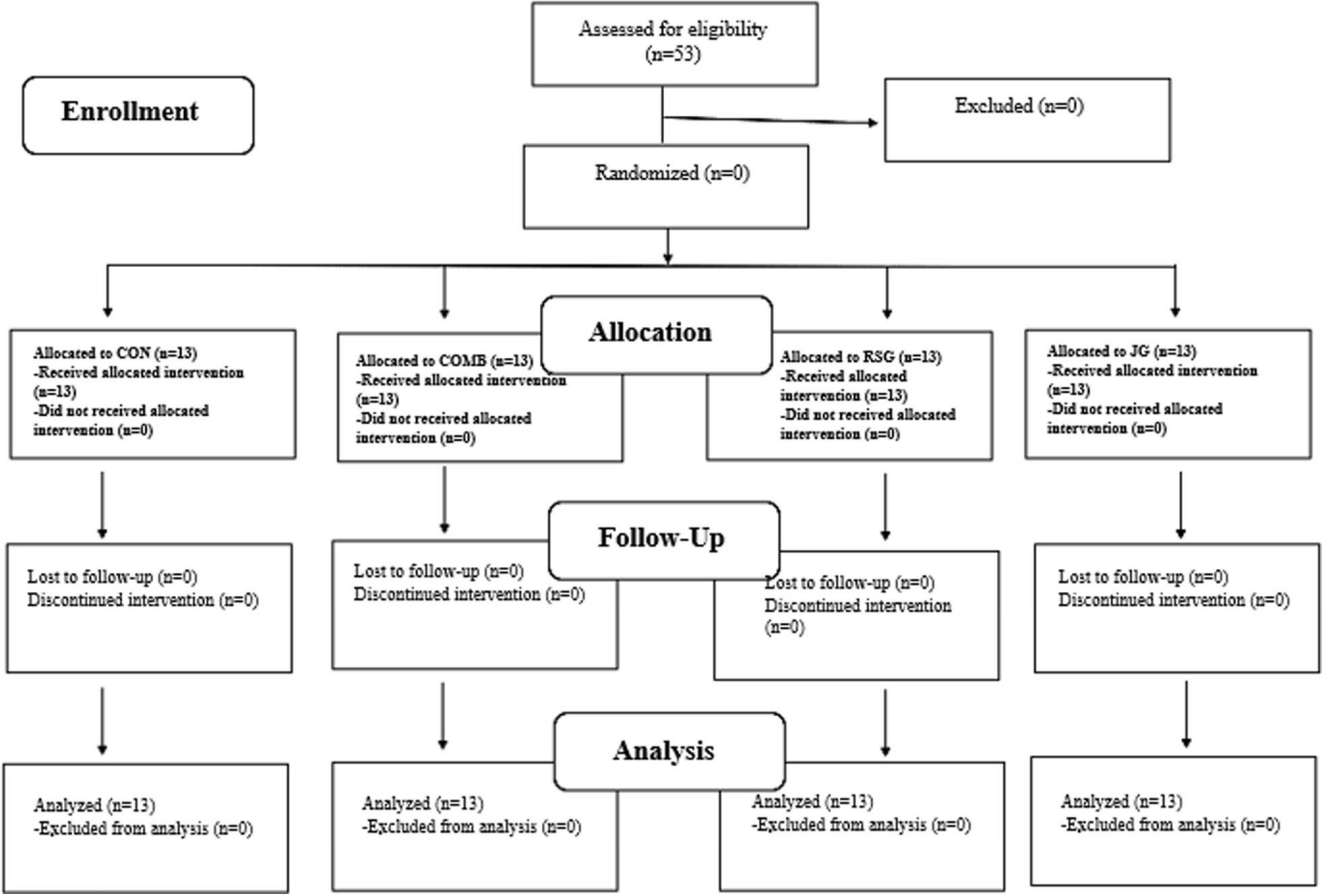
Flow chart of the progress through the phases of the study according to the CONSORT statements

The current Convenience sampling was used in this study. Fifty-two professional basketball male players from three different teams of the Tunisian first division participated in this study. Characteristics of the study population are described in Table 1. During the experimental period the three teams were of the same level and similarly ranked in the championship. For the three teams, the players had similar training experience (11.4 ± 3.6 years) and weekly practice load (≈ 9 h). Figure 1 shows a flow chart of the study design. With reference to the study of Hamammi et al. [22] an a priori power analysis [23] with an assumed Type I error of 0.01 and a type II error rate of 0.10 (90% statistical power) was conducted for results in the Y-balance test as a proxy of dynamic balance and revealed that 52 persons would be sufficient to observe a medium group × test interaction effect. All participants were eligible for inclusion in this study because they had no history of musculoskeletal, neurological or orthopedic disorders that might have affected their ability to perform physical fitness tests and to participate in the training interventions. Players were randomly assigned to drop jump (DJ) group (JG; n = 13), specific multidirectional repeated sprint (MRSA) group (RSG; n = 13), combined training (COMB; n = 13) group and an active control group (CON; n = 13). The overall adherence for the four groups was 97.68%.

**Table 1:** Anthropometric characteristics of the participating basketball players

Groups	Age (years)	Height (cm)	Body mass (kg)
JG (n = 13)	26.02 ± 2.37	194.31 ± 5.04	87 ± 6.95
RSG (n = 13)	25.75 ± 1.76	196.06 ± 4.45	82.50 ± 5.90
COMB (n = 13)	26.10 ± 1.82	192.77 ± 6.02	87.43 ± 4.23
CON (n = 13)	26.35 ± 2.11	197.01 ± 3.98	85.20 ± 3.12

Data are reported as means and standard deviations. JG: jump group; RSG: Repeated sprint group; CON: Control group; BMI: Body mass index

This study was conducted during the competitive season, and it was approved by the Clinical Research Ethics Committee of the High Institute of Sports and Physical Education of Kef, University of Jendouba, Kef, Tunisia (Approval No. 3/2018). The experimental protocol was conducted according to the latest version of the Declaration of Helsinki. All participants provided their written informed consent before study participation.

### Procedures

Players were familiarized with all tests and procedures before the start of the experimental protocol. The tests ‘reliability was verified separately during the familiarization period separated by 1 week. To allow sufficient recovery before testing, the last training session was scheduled 48 h prior to testing. To minimize any effects of diurnal variations, the two testing sessions were conducted ± 2 h of the same time of day. Players were instructed to wear the same footwear during all testing sessions [24].

Training sessions started with a 15-min warm-up followed by technical and tactical drills based on basic basketball movements (i.e., offensive, ready stance, running, CoD, linear sprint, stopping, pivoting, and jumping exercises) [7], specific basketball movements (triple threat position, pivot, face up or one- and two-phase stop), basketball technique fundamentals (dribbling, passing, and shooting), basic defensive movements (defensive stance, defensive slide, denial defense, and box-out) and a simulated game at the end of every session [7], which were identical between groups. The three experimental groups completed the same training volume (~ 90 min per session) over the course of the study.

Before (T1) and after (T2) the intervention, the players performed four testing sessions distributed as follows: the first testing session was devoted to vertical jump tests, the second testing session was devoted to CoD and FJT tests, the third testing session was devoted to the balance tests, the fourth testing session was devoted to the repeated sprint test. The testing sessions were separated with 48 h and the tests during the same testing session were performed randomly. [7, 14].

### Training program interventions

The three groups JG, RSG and COMB completed 8 weeks of their respective training programs with a frequency of two sessions per week. To ensure equal training volume between experimental groups, the volume (training weeks, sets, repetitions, and duration) of work during training was matched between groups [17]. Each training session consisted of the following sequences: (1) briefing with coaches and organization of the training session (10 min); (2) warm-up (15 min consisting of 5 min of low-intensity running, 5 min of dynamic stretching, and 5 min of skipping exercises); (3) the exercise intervention (DJ or MRSA or combined training) (20 min); (4) technical/tactical exercises to get prepared for the weekend match (30 min); (5) cool-down consisting of light running (10 min) [7]. Overall, a single training session lasted 90 min.

- DJ training program.

**Fig. 2:**
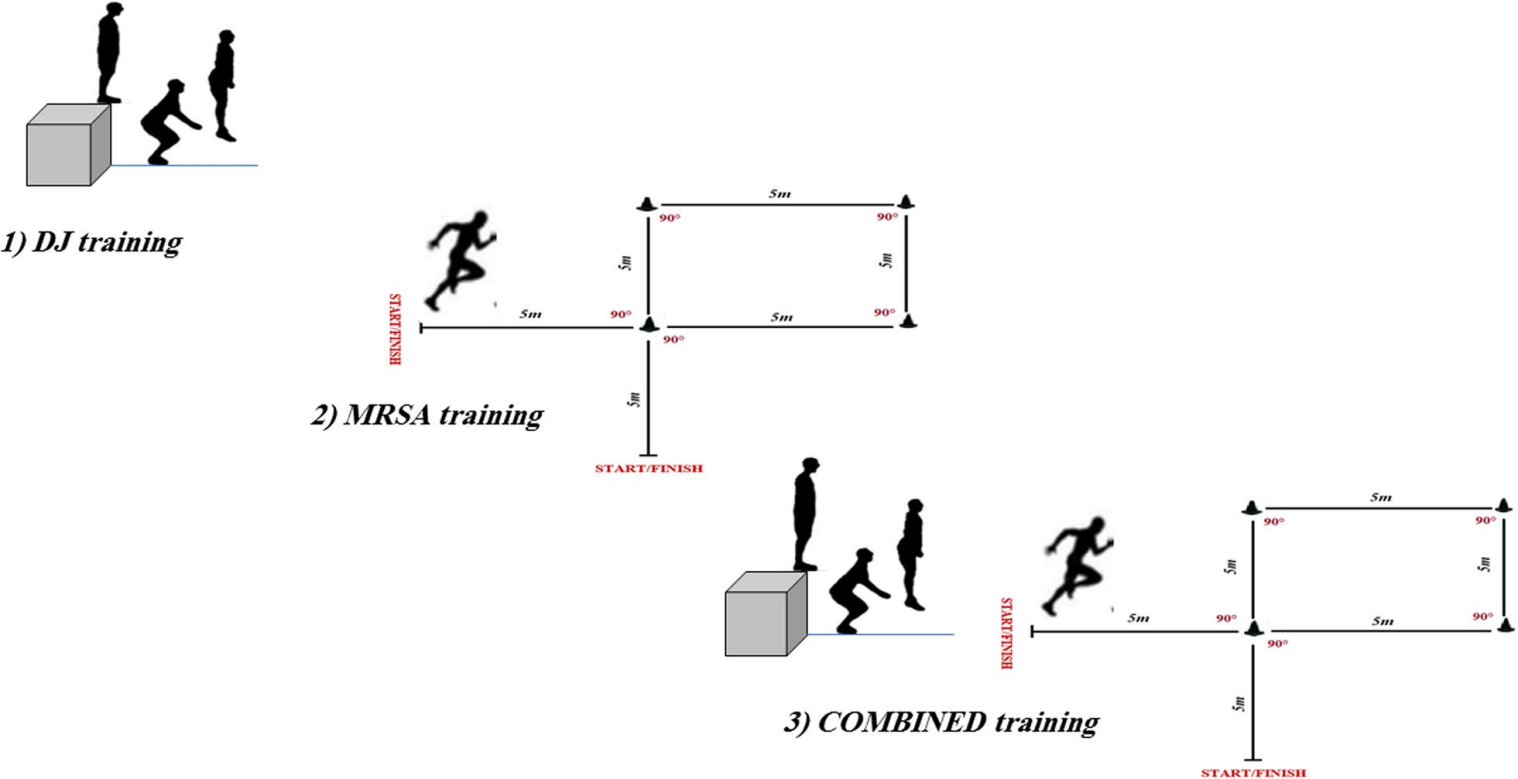
Exercise used during the training program for the JG, RSG and COMB

-Training intervention consisted of DJ performed from (50 cm) box. The jumps consisted of 3 sets of 10 repetitions during the first month and 3 sets of 12 repetitions during the second month. Recovery times between repetitions and sets were 40 s and 3 min, respectively (Fig. 2).

- MRSA training program.
  Training intervention consisted of 3 sets with 8 repetitions during the first month and 3 sets with 10 repetitions during the second month. Each repetition consisted of 30-m (6 × 5 m) sprint distances at maximal intensity (including 90° CoD) with 20 s of passive recovery between the repetitions and a 4 min rest between sets [7] (Fig. 2).
- Combined training program.
  Training intervention consisted of 3 sets of 8 repetitions during the first month [one DJ combined with one 30 m sprint (6 × 5 m) at maximal intensity (including 90° CoD)] and 3 sets of 10 repetitions during the second month. 20 s of passive recovery between the repetitions and a 4 min rest between sets (Fig. 2).

Each group was supervised by a professional strength and conditioning coach. Participants were encouraged to jump and sprint at maximal effort during each repetition.

### Training load monitoring

**Fig. 3:**
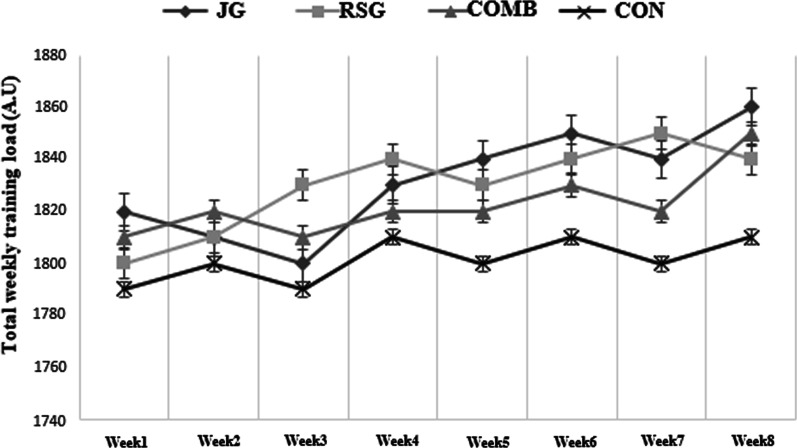
Total weekly training load during the experimental period for the JG, RSG, COMB and CON. **NB: The excel database is represented in the Additional file 1

To determine whether the participants’ global training load remained consistent through the study, the session rating of perceived exertion (RPE) training score was taken following each session. About 30-min after training sessions subjects were asked to rate the global intensity of the entire workout session using the category ratio-10 RPE scale according to the methods described by Foster et al. [25]. A daily training intensity was created by multiplying the training duration (minutes) by the session RPE. The weekly training load was determined by summing the daily training loads for each athlete during each week (Fig. 3).

### Measures

#### Anthropometrics and maximal oxygen consumption

Body mass (kg) was measured with an electronic scale (Pharo, 200 Analytic, Germany) and height (m) with a portable stadiometer (Seca, Maresten, UK). Maximal oxygen consumption (VO2max) was estimated using the 20-m shuttle run test according to the equation of Léger and Gadoury [26].

#### Stork static balance test

The stork balance test was utilized to assess static balance [19]. Subjects stood with their opposite foot against the inside of the supporting knee with both hands on the hips. On the “go” signal, subjects raised the heel from the floor and held this position for as long as possible. The test was terminated when the heel of the supporting leg touched the ground, or the foot moved away from the kneecap. The test was timed using a stopwatch. The test was performed three times, interspaced by 2 min. The best of the trials was used for further data treatment.

#### Y-balance test

The Y-balance protocol was similar to that described previously, and has a high reliability [27]. Reach directions were evaluated by affixing tape measures to the floor, one oriented anteriorly, and the other two running at 135◦ in the posterior-medial and posterior-lateral directions. All testing was conducted barefoot. Subjects stood on the dominant leg, with the most distal aspect of their great toe at the center of the grid. They then reached in the specified direction, while maintaining a single-limb stance. Tests were classified as invalid if the participants (1) did not touch the line with the reach foot while maintaining weight bearing on the stance leg, (2) lifted the stance foot from the center grid, (3) lost balance at any point during the trial, (4) did not maintain start and return positions for one full second, or (5) touched the reach foot down to gain support. The average maximum reach across the three directions (normalized for leg length) was calculated as a composite score for each subject Gribble and Hertel [28]. After a demonstration, the participant completed four practice trials in each direction. Following a 2-min rest period, three definitive trials were made in each direction. The best of trials was used for further data treatment.

#### T: change of direction test

The CoD *T* test is a valid test to evaluate CoD performances in basketball as it includes forward, lateral, and backward running over short distances [29]. Two trials were completed, and the fastest trial was taken for further analysis. Times were recorded to the nearest 0.01 s using an electronic timing system (Brower Timing Systems, Salt Lake City, UT, USA) placed 0.4 m above the ground. The test was performed two times, interspaced by 2 min. The best of trials was used for further data treatment.

#### Intensive repeated sprint ability test (IRSA_5COD_)

The IRSA5_COD_ was used and validated to assess players’ ability to cope with the intermittent demands of basketball [30]. This test consisted of 10 × 30-m shuttle sprints following a T shape, with three changes of direction of 180◦and two changes of direction of 90°, separated by 30 s of recovery. The COD occurred after each 5-m of running. The fatigue index (FI) was calculated using the Fitzsimons et al. [31] formula: (100 × (TT/(BT × 10)) − 100), where TT corresponds to total time (s) and BT to best time (s). The time for each attempt was recorded with photocells with an accuracy of 1 m (Brower timing system, Salt Lake City, UT, USA).

During the test, heart rate frequency was continuously recorded using a cardio-frequency monitor (Polar Electory, Kempte, Finland). The rating of perceived exertion (RPE) was assessed immediately following each IRSA_5COD_ test using a Borg's CR-10 scale [32]. Players were familiarized with this scale, which was regularly used during the season. The maximal blood lactate concentration (mmol L^−1^) was measured from capillary blood samples obtained from the earlobe at the 3rd min after the end of the IRSA_5COD_ test [33]. The blood sample was immediately analyzed using a portable lactate analyzer (Arkray Lactate Pro LT-1710 Kyoto, Japan) previously calibrated following the manufacturer’s instructions [34].

#### Vertical jump tests

The vertical jumps height was evaluated using an optoelectrical system (Opto-Jump Microgate, Italy). Jump height was calculated according to the following equation: jump height = 1/8 × g × t 2, where g is the acceleration due to gravity and t is the flight time [35]. Players performed the countermovement (CMJ) and the squat jumps (SJ) according to previously described protocols [36]. To assess interlimb asymmetry, a drop jump with one leg was also performed. The athlete started the movement standing upon the top of a 30 cm box. At the evaluator’s command. The asymmetry index using the following formula: (Highest performing limb–Lowest performing limb/Highest performing limb) × 100 [37]. Jump tests in the following order (counter movement jump (CMJ), squat jump (SJ) and single leg drop jump (DJ) (right/left)). All jump tests were performed three times, interspaced by 2 min. The best of trials was used for further data treatment.

#### Five-time jump test (FJT)

The FJT test is a practical and valid test and is often used as a proxy for lower limbs muscle power [38]. At the beginning of the test and after the fifth jump, feet are in parallel position. FJT performance was recorded in meters (m) to the nearest cm. Participants performed two trials and the best trial was used for further analyses. The test was performed two times, interspaced by 2 min. The best of trials was used for further data treatment.

#### Statistical analyses

All the data were presented as means and standard deviations (SD). The Shapiro Wilk test identified all variables as normally distributed. Baseline between group differences were computed using one-way ANOVA.

The effects of training were evaluated using a 4 (groups: DJ, RSG, COMB and CON) × 2 (time: Pre-test, Post-test) mixed model ANOVA. If a statistically significant interaction effect was found, Bonferroni corrected post-hoc tests were calculated.

Additionally, effect sizes (ES) were determined from ANOVA output by converting partial eta-squared to Cohen’s d. In addition, within-group ES were computed using the following equation: ES = (mean post − mean pre)/SD [39]. Following Hopkins et al. [40], ES were considered trivial (< 0.2), small (0.2 to < 0.6), moderate (0.6 to < 1.2), large (1.2 to < 2.0) and very large (2.0 to 4.0). Additionally, intraclass correlation coefficients (ICC) and coefficients of variation (CV) were computed to assess relative and absolute test–retest reliability (see Table 2). ICCs were classified as ICC < 0.50 weak, 0.50 to 0.79 moderate, and ≥ 0.80 strong. The level of significance was set at *p* < 0.05. All statistical analyses were computed using SPSS for Windows, version 20.0 (SPSS Inc., Chicago) (Table 3).

**Table 2:** Weekly training program during the experimental period for intervention and control groups

Days	Training program for intervention groups (JG, RSG and COMB)	Training program for the active control group
Monday	Warm up, 15 min Specific basketball fundamental training, 15 min Moderate intensity mid-range and 3 point shot exercises, 20 min Free throw shooting, 10 min Technical/tactical training, 25 min	Warm up, 15 min Specific basketball fundamental training, 15 min Moderate intensity mid-range and 3 point shot exercises, 20 min Free throw shooting, 10 min Technical/tactical training, 25 min
Tuesday	Warm up, 15 min The training intervention (DJ or MRSA or COMB) 20 min Free throw shooting, 10 min Moderate intensity mid-range and three point shot exercises, 20 min Technical/tactical training, 25 min	Warm up, 15 min The regular strength training (Lower body) 20 min Free throw shooting, 10 min Moderate intensity mid-range and 3 point shot exercises, 20 min Technical/tactical training, 25 min
Wednesday	Warm up, 15 min Specific basketball fundamental training, 10 min Ball drill transition training, 15 min The regular strength training (upper body) 20 min Three point shot exercises, 15 min Tactical training, 15 min	Warm up, 15 min Specific basketball fundamental training, 10 min Ball drill transition training, 15 min The regular strength training (upper body) 20 min Three point shot exercises, 15 min Tactical training, 15 min
Thursday	Warm up, 15 min The training intervention (DJ or MRSA or COMB) 20 min Free throw shooting, 10 min Moderate intensity mid-range and three point shot exercises, 20 min Technical/Tactical training, 25 min	Warm up, 15 min The regular strength training (lower body) 20 min Free throw shooting, 10 min Moderate intensity mid-range and 3 point shot exercises, 20 min Technical/Tactical training, 25 min
Friday	Warm up, 15 min Free throw shooting, 15 min Low intensity 3pts shooting exercises, 30 min Tactical training, 15 min Free throw shooting, 10 min	Warm up, 15 min Free throw shooting, 15 min Low intensity 3pts shooting exercises, 30 min Tactical training, 15 min Free throw shooting, 10 min
Saturday	Match	Match
Sunday	Recovery	Recovery

**Table 3:** Intraclass correlation coefficients (ICCs) for relative reliability and coefficients of variation for absolute reliability of the applied physical fitness tests

Measures	ICC	95% CI	% CV
SBT			
R	0.88	0.81–0.93	3.2
L	0.86	0.80–0.90	3.1
YBT			
R			
Ant	0.96	0.86–0.98	5.2
Post/Md	0.97	0.84–0.98	5.3
Post/Lat	0.97	0.85–0.99	5.1
L			
Ant	0.96	0.86–0.98	5.6
Post/Md	0.95	0.87–0.97	5.4
Post/Lat	0.97	0.85–0.99	5.2
*T* test	0.98	0.90–0.99	2.8
IRSA_5COD_			
TT	0.96	0.90–0.97	2.3
BT	0.97	0.89–0.99	2.2
CMJ	0.98	0.93–0.99	3.7
SJ	0.97	0.91–0.98	3.5
DJ			
R	0.96	0.88–0.98	4.1
L	0.95	0.87–0.97	4.4
FJT	0.98	0.86–0.97	3.2

*ICC* intraclass correlation coefficient, *CI* confidence interval, *CV* coefficient of variation (%). *IRSA*_*5COD*_ repeated sprint ability test with five CoDs

## Results

All players completed the study according to the previously described methodology. No injuries occurred over the course of the study. Adherence rates were 97.6% for JG, 97.8% for RSG and COMB. The average playing time per game was 27.5 ± 1.8 min for JG, 27.7 ± 1.5 min for RSG and 27.8 ± 1.7. No statistically significant between group differences were observed for these measures. In addition, no significant between-group baseline differences were found for any of the analysed parameters (see Tables 4, 5).

**Table 4:** Intensive repeated sprint ability test performances and physiological parameters determined before (pre-test) and after (post-test) the training program

Groups													*p* values (effect size)		
Variables	JG (n = 13)			RSG (n = 13)			COMB (n = 13)			CON (n = 13)			Time	Group	Group × Time
	Pre Test	Post Test	Δ%	Pre Test	Post Test	Δ%	Pre Test	Post Test	Δ%	Pre Test	Post Test	Δ%			
TT (s)	83.42 ± 0.24	83.08 ± 0.39	− 0.41 ± 0.41	83.55 ± 0.32	82.85 ± 0.44	− 0.84 ± 0.40	83.58 ± 0.31	82.92 ± 0.28	− 0.79 ± 0.09	83.38 ± 0.09	83.35 ± 0.08	− 0.05 ± 0.08	0.000 (0.92)	0.40 (0.08)	0.000 (0.64)
BT (s)	8.19 ± 0.05	8.10 ± 0.03	− 0.79 ± 0.54	8.22 ± 0.09	8.05 ± 0.04	− 2 ± 0.97	8.24 ± 0.08	8.07 ± 0.04	− 2.05 ± 0.70	8.17 ± 0.10	8.16 ± 0.08	− 0.12 ± 0.39	0.000 (0.96)	0.56 (0.05)	0.000 (0.64)
FI (%)	1.91 ± 0.64	2.48 ± 0.56	49.05 ± 67.08	1.65 ± 0.83	2.86 ± 0.58	288.7 ± 703.07	1.50 ± 0.89	2.81 ± 0.40	246.97 ± 492.75	2.03 ± 1.24	2.10 ± 1.05	9.85 ± 41.05	0.000 (0.87)	0.51 (0.53)	0.03 (0.27)
HR (beat/min)	187.30 ± 2.39	137.85 ± 2.34	0.02 ± 0.21	187.87 ± 2.71	178.67 ± 2.66	− 0.10 ± 0.59	188.59 ± 1.63	188.66 ± 1.58	0.04 ± 0.22	188.82 ± 1.80	188.84 ± 1.77	0.01 ± 0.09	0.87 (0.002)	030. (0.10)	0.55 (0.04)
[Lac] (mmol/l)	7.77 ± 2.29	8.12 ± 186	6.85 ± 15.80	8.76 ± 1.41	9.35 ± 1.60	10.21 ± 29.34	8.25 ± 156	8.34 ± 1.68	1 ± 3.81	9.55 ± 1.42	9.53 ± 1.18	0.35 ± 4.19	0.22 (0.13)	0.09 (0.18)	0.04 (0.65)
RPE	6.69 ± 0.63	6.92 ± 0.76	3.85 ± 11.02	6.92 ± 0.46	7.38 ± 0.65	7.65 ± 15.03	7.08 ± 0.76	6.92 ± 0.79	− 1.10 ± 13.09	7.23 ± 0.73	7.24 ± 0.60	1.05 ± 13.68	0.40 (0.06)	0.20 (0.12)	0.27 (0.10)

Data are reported as means and standard deviations. *TT* total time, *BT* best time, *FI* fatigue index; *HR* heart rate, *[Lac]* Lactate concentration, *RPE* rating of perceived exertion

**Table 5:** Body balance, change-of-directions and Jump performances determined before (pre-test) and after (post-test) training program

Groups													*p* values (effect size)		
Variables	JG (n = 13)			RSG (n = 13)			COMB (n = 13)			CON (n = 13)			Time	Group	Group × Time
	Pre Test	Post Test	Δ%	Pre Test	Post Test	Δ%	Pre Test	Post Test	Δ%	Pre Test	Post Test	Δ%			
SBT (s)															
R	19.69 ± 1.06	21.76 ± 1.89	10.53 ± 6.58	19.09 ± 0.75	23.39 ± 1.36	22.60 ± 6.91	19.05 ± 1.09	23.16 ± 0.92	21.97 ± 9.10	19.55 ± 0.50	19.86 ± 0.74	1.63 ± 3.30	0.000 (0.94)	0.011 (0.34)	0.000 (0.74)
L	19.41 ± 1.28	21.48 ± 1.61	10.69 ± 3.88	18.84 ± 0.72	23.09 ± 1.24	22.67 ± 6.77	19.29 ± 0.87	23.02 ± 1.17	19.57 ± 8.59	19.38 ± 0.83	19.58 ± 1.31	1.02 ± 5.15	0.000 (0.95)	0.002 (0.38)	0.000 (0.76)
YBT (cm)															
R															
Ant	88.07 ± 3.01	89.76 ± 3.96	1.91 ± 2.25	88.30 ± 2.75	95 ± 2.91	7.59 ± 1.63	88.92 ± 3.35	96.92 ± 3.04	9.03 ± 1.71	88.69 ± 2.71	87.54 ± 6.01	− 1.23 ± 7.12	0.000 (0.84)	0.001 (0.73)	0.000 (0.62)
Post/Md	97.54 ± 2.57	101.23 ± 2.59	3.80 ± 1.60	97.85 ± 5.68	107 ± 5.58	9.42 ± 2.48	98.07 ± 2.25	108.69 ± 3.17	10.83 ± 3.01	97.46 ± 2.54	97.69 ± 3.01	0.24 ± 1.63	0.000 (0.98)	0.003 (0.38)	0.000 (0.87)
Post/Lat	59.38 ± 2.47	63.07 ± 2.47	6.24 ± 1.79	59.31 ± 2.43	64.54 ± 1.20	8.95 ± 3.96	58.69 ± 1.18	64.84 ± 1.57	10.51 ± 3.05	58.31 ± 1.18	58.85 ± 1.28	0.94 ± 2.30	0.000 (0.97)	0.000 (0.93)	0.000 (0.86)
L															
Ant	88.76 ± 2.01	92.54 ± 1.94	4.25 ± 0.84	89.15 ± 4.07	95.92 ± 3.23	7.66 ± 1.98	88.85 ± 3.08	97.07 ± 2.78	9.31 ± 2.32	88.54 ± 1.90	88.76 ± 1.96	0.27 ± 1.55	0.000 (0.98)	0.001 (0.43)	0.000 (0.86)
Post/Md	99.92 ± 2.21	102.62 ± 2.75	2.70 ± 1.90	100.31 ± 2.25	109 ± 3.05	8.67 ± 2.06	100.85 ± 2.82	110.77 ± 3.32	9.85 ± 2.09	100.62 ± 1.71	100.92 ± 1.98	0.31 ± 0.94	0.000 (0.97)	0.000 (0.61)	0.000 (0.89)
Post/Lat	58.61 ± 1.26	61.62 ± 2.06	5.16 ± 4.15	58.85 ± 1.95	64.23 ± 1.42	9.25 ± 4.03	58.31 ± 2.46	64.54 ± 1.85	10.80 ± 3.89	58.53 ± 1.81	59.54 ± 1.51	1.74 ± 1.77	0.000 (0.91)	0.001 (0.42)	0.000 (0.66)
*T* test (s)	6.66 ± 0.07	6.60 ± 0.06	− 0.96 ± 0.75	6.69 ± 0.07	6.55 ± 0.05	− 1.98 ± 0.80	6.65 ± 0.07	6.48 ± 0.05	− 2.49 ± 0.74	6.67 ± 0.05	6.65 ± 0.04	− 0.32 ± 0.87	0.000 (0.94)	0.001 (0.42)	0.000 (0.61)
SJ (cm)	36.77 ± 2.09	41.23 ± 2.98	12.24 ± 7.39	36.54 ± 2.99	38.62 ± 2.26	5.94 ± 4.47	37.08 ± 1.94	39.07 ± 2.18	5.43 ± 3.58	37.62 ± 1.98	37.69 ± 2.02	0.27 ± 4.08	0.000 (0.88)	0.27 (0.10)	0.000 (0.52)
CMJ (cm)	38.77 ± 5.88	43.76 ± 5.63	13.40 ± 5.65	39.85 ± 6.44	42 ± 5.87	5.78 ± 3.51	38.38 ± 4.84	41.08 ± 4.05	7.47 ± 5.27	38 ± 2.55	38.15 ± 2.79	0.49 ± 4.92	0.000 (0.91)	0.34 (0.09)	0.000 (0.63)
DJ (cm)															
R	14,70 ± 1.85	17.08 ± 1.55	16.98 ± 9.65	14.84 ± 2.41	16.23 ± 2.09	9.97 ± 6.50	14.31 ± 1.32	15.46 ± 1.27	8.31 ± 5.44	14.69 ± 1.80	14.77 ± 1.64	0.95 ± 7.71	0.000 (0.87)	0.33 (0.09)	0.000 (0.52)
L	14.76 ± 2.55	17 ± 2.38	15.77 ± 6.19	14.08 ± 2.25	14.92 ± 1.55	7.21 ± 9.98	14.38 ± 1.39	15.23 ± 1.42	6.03 ± 5.10	14.92 ± 1.55	15.31 ± 1.44	3.07 ± 9.53	0.000 (0.80)	0.24 (0.11)	0.002 (0.38)
ASI	13.84 ± 5.81	9 ± 6.25	− 34.32 ± 36.79	13.82 ± 5.76	12.88 ± 9.38	7.01 ± 116.04	9.71 ± 5.81	8.51 ± 5.18	160.17 ± 541.01	12.89 ± 5.83	10.89 ± 5.74	5.10 ± 71.92	0.08 (0.0.24)	0.16 (0.13)	0.53 (0.05)
FJT (m)	8 ± 0.39	8.24 ± 0.38	3.04 ± 0.76	8.05 ± 0.49	8.13 ± 0.47	1.05 ± 0.51	8.01 ± 0.53	8.14 ± 0.54	1.59 ± 1.17	8.19 ± 0.60	8.21 ± 0.59	0.16 ± 0.42	0.000 (0.94)	0.89 (0.01)	0.000 (0.73)

Data are reported as means and standard deviations. *SBT* Stork balance test, *YBT* Y-balance test, *R* right leg, *L* left leg, *Ant* anterior, *Post/Md* postero-medial, *Post/Lat* *T* test: CoD T test; *SJ* squad jump test, *CMJ* countermovement jump test; *DJ* single leg drop jump test, *ASI* asymmetry index, *FJT* five jump test

•Reliability.

Table 2 illustrates ICCs for relative reliability and CV for absolute reliability of the applied physical fitness tests. Reliability measures ICCs ranged from 0.86 to 0.99 and CV ranged from 2.2 to 5.6 for all tests.

•Main Effects.

All measures displayed significant main effect, (medium/small) magnitude (effect size) improvements for time (post-test > pre-test) (Tables 4, 5) except for Physiological parameters for (IRSA_5COD_). Significant main effects for group were evident with the three groups except for jump performances (Table 4). With each of these measures the control group was significantly different from the JG, RSG and COMB groups.

•Interactions.

### Body balance

Significant group x time was observed for SBT test performance on both legs [right leg (*p* < 0.001, ES = 0.74, moderate), left leg (*p* < 0.001, ES = 0.76, moderate)]. Bonferroni corrected post-hoc test for right leg revealed significant pre-to-post improvements for JG, RSG and COMB with a better improvement in favor of RSG and COMB [(10.53%, *p* < 0.001, ES = 0.36, small), (22.60%, *p* < 0.001, ES = 0.35, small), (21.97%, *p* < 0.001, ES = 0.42, small), respectively]. Moreover, Bonferroni corrected post-hoc test revealed significant pre-to-post improvements for JG, RSG and COMB with a better improvement in favor of RSG [(10.69%, *p* < 0.001, ES = 0.21, Small), (22.67%, *p* < 0.001, ES = 0.33, small), (19.57%, *p* < 0.001, ES = 0.43, small), respectively].

Significant group x time interaction was observed for YBT test performance for the right leg support (anterior: *p* < 0.001, ES = 0.62, moderate; posteromedial: *p* < 0.001, ES = 0.87, moderate; posterolateral: *p* < 0.001, ES = 0.76, moderate). Bonferroni corrected post-hoc test revealed significant pre-to-post improvements for JG, RSG and COMB with a better improvement in favor of RSG and COMB [(JG: anterior: 1.91%, *p* = 0.009, ES = 0.55, small; posteromedial: 3.80%, *p* < 0.001, ES = 0.42, small; posterolateral: 6.24%, *p* < 0.001, ES = 0.29, small); RSG: anterior: 7.59%, *p* < 0.001, ES = 0.38, small; posteromedial: 9.42%, *p* < 0.001, ES = 0.63, moderate; posterolateral: 8.95%, *p* < 0.001, ES = 0.59, small); (COMB: anterior: 9.03%, *p* < 0.001, ES = 0.38, small; posteromedial: 10.83%, *p* < 0.001, ES = 0.68, moderate; posterolateral: 10.51%, *p* < 0.001, ES = 0.48, small); respectively]. For the left support leg, we found similar results (anterior: *p* < 0.001, ES = 0.86, moderate; posteromedial: *p* < 0.001, ES = 0.89, moderate; posterolateral: *p* < 0.001, ES = 0.66, moderate). Bonferroni corrected post-hoc test revealed significant pre-to-post improvements for JG, RSG and COMB with a better improvement in favor of RSG and COMB[(JG: anterior: 4.25%, *p* < 0.001, ES = 0.20, small; posteromedial: 2.70%, *p* < 0.001, ES = 0.52, small; posterolateral: 5.16%, *p* = 0.001, ES = 0.66, moderate); RSG: anterior: 7.66%, *p* < 0.001, ES = 0.44, small; posteromedial: 8.67%, *p* < 0.001, ES = 0.57, small; posterolateral: 9.25%, *p* < 0.001, ES = 0.62, moderate); (COMB: anterior: 9.31%, *p* < 0.001, ES = 0.52, small; posteromedial: 9.85%, *p* < 0.001, ES = 0.57, small; posterolateral: 10.80%, *p* < 0.001, ES = 0.56, small); respectively].

### Change of direction

Significant group × time interaction was observed for *T* test (*p* < 0.001, ES = 0.61, moderate). Bonferroni corrected post-hoc test revealed significant pre-to-post improvements for JG, RSG and COMB with a better improvement in favor of COMB [(− 0.96%; *p* = 0.001, ES = 0.01, trivial), (− 1.98%, *p* < 0.001, ES = 0.02, trivial), (− 2.49%, *p* < 0.001, ES = 0.05, trivial), respectively].

### Intensive repeated sprint ability test

Significant group × time interaction was observed for TT (*p* < 0.001, ES = 0.64, moderate). Bonferroni corrected post-hoc test revealed significant pre-to-post improvements for JG, RSG and COMB with a better improvement in favor of RSG [(− 0.41%, *p* = 0.003, ES = 0.10, trivial), (− 0.84%, *p* < 0.001, ES = 0. 10, small), (− 0.79%, *p* < 0.001, ES = 0. 21, small), respectively].

Significant group × time interaction was observed for BT (*p* < 0.001, ES = 0.57, moderate). Bonferroni corrected post-hoc test revealed significant pre-to-post improvements for JG, RSG and COMB with a better improvement in favor of RSG and COMB [(− 0.79%, *p* < 0.001, ES = 0.01, trivial), (− 2%, *p* < 0.001, ES = 0.02, trivial), (− 2.05% *p* < 0.001, ES = 0.02, trivial), respectively].

### Vertical and horizontal jump

Significant group × time interaction was observed for CMJ (*p* < 0.001, ES = 0.52, small). Bonferroni corrected post-hoc test revealed significant pre-to-post improvements for JG, RSG and COMB with a better improvement in favor of JG [(13.40%; *p* < 0.001, ES = 0.45, small), (5.78%, *p* < 0.001, ES = 0.30, small), (7.47%, *p* < 0.001, ES = 0.46, small), respectively].

Significant group × time interaction was observed for SJ (*p* < 0.001, ES = 0.63, moderate). Bonferroni corrected post-hoc test revealed significant pre-to-post improvements for JG, RSG and COMB with a better improvement in favor of JG [(12.24%, *p* < 0.001, ES = 0.70, moderate), (5.94%, *p* < 0.001, ES = 0.38, Small), (5.43%, *p* < 0.001, ES = 0.27, small), respectively].

Significant group × time was observed for DJ test performance on both legs [right leg (*p* < 0.001, ES = 0.52, small), left leg (*p* = 0.002, ES = 0.38, small)]. Bonferroni corrected post-hoc test for right leg revealed significant pre-to-post improvements for JG, RSG and COMB with a better improvement in favor of JG [(16.98%, *p* < 0.001, ES = 0.35, small), (9.97%, *p* < 0.001, ES = 0.21, small), (8.31%, *p* < 0.001, ES = 0.19, small), respectively]. Moreover, Bonferroni corrected post-hoc test revealed significant pre-to-post improvements for JG, RSG and COMB with a better improvement in favor of JG [(15.77%, *p* < 0.001, ES = 0.20, small), (7.21%, *p* = 0.035, ES = 0.36, small), (6.03% *p* = 0.001, ES = 0.19, small), respectively].

Significant group × time interaction was observed for FJT (*p* < 0.001, ES = 0.73, moderate). Bonferroni corrected post-hoc test revealed significant pre-to-post improvements for JG, RSG and COMB with a better improvement in favor of JG [(3.04%, *p* < 0.001, ES = 0.02, trivial), (1.05% *p* < 0.001, ES = 0.01, trivial), (1.59%, *p* < 0.001, ES = 0.03, trivial), respectively].

## Discussion

The main aim of this study was to examine the contributions of 8-week combined versus single-mode training programs based on DJ and MRSA on RSA performances, body balance and lower limbs power in professional basketball male players. In general, the findings of the present study showed that the three training interventions lead to a significant improvement compared to the CON. Additionally, a greater improvement was recorded in favor of RSG and COMB compared to JG for body balance, CoD and RSA. Moreover, better improvement in CMJ, SJ and single leg DJ was recorded in favor of JG compared to the RSG. A better CoD improvement in favor of COMB compared to the RSG was reported. Therefore, the data of the present study partially confirmed our hypothesis.

### Drop jump training effects on selected physical fitness tests

8-week of DJ training significantly improved body balance, RSA performances, CoD and jump performances. Improvements in balance performances following DJ training in the present study were in line with previous studies incorporating vertical jumping exercises [8, 41, 42]. In fact, the improvement in balance performance may be related to improved co-contraction of lower body muscles [43] and/or to changes in proprioception and neuromuscular control [44]. Concerning the significant increases in IRSA_5COD_ performances (TT and BT), the findings of the present study may be explained by the change in explosive performance after a plyometric training program which may contribute to improvement during RSA test with CoD [45]. Moreover, several earlier plyometric studies have shown that this type of exercises can enhance sprinting performance in basketball and soccer players [15, 46].

Eccentric strength is an important determinant of deceleration ability during CoD actions [47]. The higher inertia accumulated in the braking phase during plyometric training may have contributed to increases in eccentric workload and, therefore, larger strength improvements [48]. Moreover, previous studies have explained the improvements in CoD performance with the interaction of several neuromuscular adaptations (i.e., higher efficiency of SSC), and muscle activation strategies that promote improved inter- and intra-muscular coordination [49]. Finally, our findings were in accordance with the existing literature, which has reported improved jump performance after specific plyometric programs including DJ [12]. Improved jump performance as a result of plyometric training may be partially attributable to improved motor recruitment, the elastic benefits to SSC, and/or a muscle typology shifts [49].

### Specific multidirectional repeated sprint effects on selected physical fitness tests

The MRSA training intervention significantly improved body balance, RSA performances (TT and BT), CoD and jump performances. For the significant improvement of body balance (Static/Dynamic), our results may be attributed to the balance challenges associated with CoD training. In this context, previous studies have reported that (rapid CoD/ training program based on CoD) has some benefits over stationary and dynamic balance since that high-speed with CoD impose frequent perturbations upon postural control which may positively affect athletic performance [11, 27, 50, 51]. In the same context, Hammami et al. [27] and Sekulic et al. [52] reported a significant correlation between CoD and body balance.

Concerning the significant improvement in TT and BT, our results are likely explained by the fact that our training design was specifically inspired from the IRSA_5COD_ test protocol and it aims primarily to improve the performance of this test. In this context, our results align with those of Attene et al. [9] in young basketball players and Buchheit et al. [45] in elite adolescent soccer players. However, the present study disagrees with Brini et al. [7]. Those authors reported no significant changes in RSA performances (TT and BT) after 12-week using the same MRSA training programs. The differences between the two studies could be attributed to fatigue accumulated at the end of the training period revealed by those authors which was witnessed by a decrease in the Testosterone/cortisol ratio [7]. Therefore, a shorter study duration (8-week) may lead to a better RSA performance and avoid overtraining. Regarding FI, the usefulness of this index for a coach is still under debate because of some reproducibility problems, moreover a better FI does not necessarily indicate better RSA performances [53, 54]. Concerning the *T* test, our results showed a significantly better CoD at the end of MRSA training intervention which was in accordance with previous investigations that demonstrated a significant improvement of CoD performance following repeated sprints training with CoD [8, 9]. Our results could be explained by the similarity between the IRSA_5COD_ and the *t *test in design and demands thus allowing to a better CoD performance.

In the present study, the jump performance significantly increased at the end of the MRSA intervention. Our findings were in line with previous studies reporting significant improvement in jump performances in basketball players completing a shorter training period including CoD repeated sprint protocol [9]. In fact, those authors explained this significant improvement by the very large correlation between sprint and jumping performance. Moreover, several studies reported that RSA training increases leg muscle explosive power, improvements in motor unit synchronization, and SSC efficiency [55].

### Combined training program effects on selected physical fitness tests

During the present investigation, the combined training intervention significantly improved body balance, RSA performances (TT and BT), CoD and jump performances. Significant improvement in body balance may be explained by the significant improvement observed following training in this study could be due to an enhancement in motor coordination [56], and the improved neuromuscular control of lower limb muscle following this type of training [57]. In this context, our findings corroborate the data of several previous studies [17, 19]. Those authors reported improved static (stork balance test) and dynamic (Y-balance test) balance performance as a result of an 8-week plyometric and change-of-direction exercise program in prepubertal male soccer players.

For the IRSA_5COD_ test_,_ the significant improvement of the TT and BT may be explained by the enhancements in explosive power through improvements in motor unit synchronization, SSC efficiency, or musculotendinous stiffness following the combined training program [45–58].

Concerning Jump performance, our results were in accordance with previous studies that reported a significant improvement [19, 59, 60]. Improved jump performance as a result of plyometric training may be partially attributable to improved motor recruitment, the elastic benefits to the SSC, and/or a muscle typology shifts [49].

### DJ versus MRSA versus combined

The findings of the present study showed a significantly better improvement recorded in favor of RSG and COMB compared to JG for body balance, CoD and RSA. Moreover, better improvement in CMJ, SJ and single leg DJ was recorded in favor of JG compared to the RSG. Only CoD showed greater improvement in favor of COMB compared to the RSG.

For the better body balance obtained in favor of the RSG and COMB, our results could be explained mainly by the nature of the MRSA and the combined mode based on CoDs protocols. In this context, several investigations reported that additional changes of directions activities lead to a greater balance and body control [27, 51]. In the same context, Jones et al. [50] reported that high-speed change of direction imposes frequent perturbations upon postural control.

Concerning the better CoD and RSA in favor of RSG and COMB compared to the DJ our results were not surprising and somewhat expected since the MRSA training protocol and the combined mode were designed and extracted from the IRSA_5COD_ test protocol firstly (by including the same CoD angles and sprint distances). Secondly, the combination of speed, jumping and CoD in the combined mode seems to have a great and specific impact in high-intensity actions better than DJ.

For the better improvements in jump performance recorded in favor DJ compared to the RSG group our results could be explained by the fact that the RSG did not perform jump exercise during 8-week. In this context, several studies reported that single mode MRSA training performed without any additional strength or plyometric training could affect jump performance [7, 61].

The differences between COMB and RSG in term of CoD performance was logical since it was admitted that MRSA training program improves CoD performance in basketball players [7] and by adding the plyometric regimen which is known to improve the eccentric strength of thigh muscles, a prevalent component in CoD during the deceleration phase of impulsive movement which may lead to a better performance.

### Limitations

Although we present a novel addition to the literature, our study has some limitations that warrant consideration. First, our study examined only professional basketball male players. Thus, future studies should extend these observations to other age groups, female players and other skill levels. Moreover, the present study was limited only by monitoring the session RPE and did not investigate other internal load responses such us: inflammatory makers, enzymes and/or testosterone ratio hormones, given that of the lack of the financial supports. Therefore, future studies are encouraged to explore authors indicator about injuries, pain, or other adverse effects that occur as a result of those training methods.

## Conclusions

The results of the present study showed that both combined and single-mode training programs based on DJ and MRSA contributed to a significant better performance in some specific basketball physical fitness parameters with better performances recorded in favor of the combined mode.

### Practical applications

The present study indicates that additional 8-week combined and single-mode training programs based on drop jump and specific multidirectional repeated sprint enhances body balance, IRSA_5COD_ performances (TT and BT), CoD and jump performances in professional basketball players with a significantly larger contribution in favor of the combined training mode. Thus, it will be practicable to incorporate this training mode into daily in-season male basketball training sessions, thus enhancing the performance potential of our players. Moreover, our finding showed the beneficial impact of the single mode training (DJ /or MRSA) in some key physical fitness parameters specific to the basketball exergy. Thus, we advise coaches and physical trainers to use also those specific single mode training depending on the objectives and the season phases (Additional file 1).

## Supplementary Information

**Additional file 1**. All additional information for trainers.

## Data Availability

The datasets generated during and analyzed during the current study are not publicly available due to confidential information about the participants but are available from the corresponding author on reasonable request at [bseifeddine15@gmail.com].

## Abbreviations

DJ: Drop jump
MRSA: Multidirectional repeated sprint
JG: Drop jump group
RSG: Repeated sprint group
COMB: Combined group
CON: Active control group
STB: The stork test
YBT: Y-balance test
IRSA_5COD_: The repeated sprint ability test with CoD
TT: Total time
BT: Best time
FI: Fatigue index
HR: Heart rate
RPE: The rating of perceived exertion
SJ: The squat jump
CMJ: Countermovement jump
FJT: The five time-jump test
CoD: Change of direction
